# Incidence of Norovirus and Other Viral Pathogens That Cause Acute Gastroenteritis (AGE) among Kaiser Permanente Member Populations in the United States, 2012–2013

**DOI:** 10.1371/journal.pone.0148395

**Published:** 2016-04-26

**Authors:** Scott P. Grytdal, Emilio DeBess, Lore E. Lee, David Blythe, Patricia Ryan, Christianne Biggs, Miriam Cameron, Mark Schmidt, Umesh D. Parashar, Aron J. Hall

**Affiliations:** 1 Division of Viral Diseases, National Center for Immunization and Respiratory Diseases, Centers for Disease Control and Prevention, Atlanta, GA, United States of America; 2 Public Health Division, Department of Human Services, Oregon Health Authority, Portland, OR, United States of America; 3 Office of Infectious Disease and Epidemiology, Maryland Department of Health and Mental Hygiene, Baltimore, MD, United States of America; 4 Kaiser Permanente of the Mid-Atlantic States, Rockville, MD, United States of America; 5 Kaiser Permanente Center for Health Research, Portland, OR, United States of America; University of Malaya, MALAYSIA

## Abstract

Noroviruses and other viral pathogens are increasingly recognized as frequent causes of acute gastroenteritis (AGE). However, few laboratory-based data are available on the incidence of AGE caused by viral pathogens in the U.S. This study examined stool specimens submitted for routine clinical diagnostics from patients enrolled in Kaiser Permanente (KP) health plans in metro Portland, OR, and the Maryland, District of Columbia, and northern Virginia geographic areas to estimate the incidence of viral enteropathogens in these populations. Over a one-year study period, participating laboratories randomly selected stools submitted for routine clinical diagnostics for inclusion in the study along with accompanying demographic and clinical data. Selected stools were tested for norovirus, rotavirus, sapovirus, and astrovirus using standardized real-time RT-PCR protocols. Each KP site provided administrative data which were used in conjunction with previously published data on healthcare utilization to extrapolate pathogen detection rates into population-based incidence rates. A total of 1,099 specimens collected during August 2012 to September 2013 were included. Mean age of patients providing stool specimens was 46 years (range: 0–98 years). Noroviruses were the most common viral pathogen identified among patients with AGE (n = 63 specimens, 6% of specimens tested). In addition, 22 (2%) of specimens were positive for rotavirus; 19 (2%) were positive for sapovirus; and 7 (1%) were positive for astrovirus. Incidence of norovirus-associated outpatient visits was 5.6 per 1,000 person-years; incidence of norovirus disease in the community was estimated to be 69.5 per 1,000 person-years. Norovirus incidence was highest among children <5 years of age (outpatient incidence = 25.6 per 1,000 person-years; community incidence = 152.2 per 1,000 person-years), followed by older adults aged >65 years (outpatient incidence = 7.8 per 1,000 person-years; community incidence = 75.8 per 1,000 person-years). Outpatient incidence rates of rotavirus, sapovirus, and astrovirus were 2.0, 1.6, 0.6 per 1,000 person-years, respectively; community incidence rates for these viruses were 23.4, 22.5, and 8.5 per 1,000 person-years, respectively. This study provides the first age-group specific laboratory-based community and outpatient incidence rates for norovirus AGE in the U.S. Norovirus was the most frequently detected viral enteropathogen across the age spectrum with the highest rates of norovirus disease observed among young children and, to a lesser extent, the elderly. These data provide a better understanding of the norovirus disease burden in the United States, including variations within different age groups, which can help inform the development, targeting, and future impacts of interventions, including vaccines.

## Background

Acute gastroenteritis (AGE), characterized by sudden onset of symptoms including diarrhea, vomiting, nausea, and abdominal pain, is a major cause of morbidity in the United States. Approximately 179 million cases of AGE are estimated to occur annually in the United States, resulting in 600,000 hospitalizations and an estimated 5,000 deaths [1, 2]. Viruses have been found to be the most common known agents of AGE. Noroviruses specifically are estimated to cause approximately 19–21 million cases of AGE annually, including 56,000–71,000 hospitalizations and 570–800 deaths [3]. With wide diversity of noroviruses in circulation and lack of persistent cross-protective immunity, on average, a U.S. resident experiences five norovirus episodes in his or her lifetime [3]. Other viruses such as rotavirus, astrovirus, and sapovirus are also important agents of AGE, especially among children. Diarrhea healthcare utilization has been dramatically reduced among children under 5 years due to rotavirus vaccination in the United States [4, 5]. The burden of AGE disease in the United States attributable to sapoviruses and astroviruses is less clear and studies have been limited to children less than 5 years of age [6] and to members of a health maintenance organization in one state [7].

Early estimates of the burden and incidence of norovirus AGE in the United States were based on extrapolations of etiologic fractions from studies in other industrialized countries [2, 8], but more recent estimates have included direct testing for viral pathogens in fecal specimens [7, 9]. Laboratory-confirmed population-based estimates of norovirus AGE in the U.S. remain limited to pediatric populations and all-ages estimates, which were not adequately powered to provide age-specific rates [7, 9, 10]. Age-stratified rates of norovirus AGE covering the full age spectrum in other industrialized countries are even rarer and have only been estimated for the United Kingdom. [11].

Direct assessments of laboratory-confirmed, age-specific incidence of norovirus AGE are needed to assess the need and potential impacts of targeted interventions. Continued development and subsequent implementation of candidate norovirus vaccines must be built upon reliable age-specific incidence estimates for the U.S. [12]. To address this need, our primary objective was to estimate age-specific incidence rates of norovirus AGE among members of Kaiser Permanente (KP) health plans in metro Portland, OR, and the Maryland, District of Columbia, and northern Virginia geographic areas from 2012–2013. Our secondary objective was to generate all-ages estimates of the incidence of other viral pathogens associated with AGE, specifically rotavirus, sapovirus, and astrovirus.

## Methods

### Study Sites and Data Collection

This study was conducted in conjunction with the Mid-Atlantic and Northwest regions within the KP health maintenance organization. The Kaiser Mid-Atlantic region consists of approximately 488,000 members and encompasses the Washington, D.C. metropolitan area, including portions of Maryland and Virginia. The Kaiser Northwest region consists of approximately 480,000 members within the metropolitan area of Portland, Oregon, including areas of northwest Oregon and southwestern Washington. Members of KP almost exclusively seek care within the organization.

Diagnostic stool specimens submitted for routine clinical testing (i.e., bacterial culture) from all outpatients who sought medical care at each KP site were randomly selected for inclusion in this study. A target minimum sample size of 511 specimens from each site was determined based on power calculations using expected values from previous studies (i.e., 5% expected norovirus prevalence, assuming that stool specimens from cases of non-bloody diarrhea of ≤3 days duration are 7 times less likely to be collected than from all cases of diarrhea) [7]. The precision of overall norovirus incidence increases when the number of specimens exceeds the targeted sample size. Only patients for whom a specimen was submitted after a clinician order for routine bacterial culture, ova and parasite test, or for the presence of *Clostridium difficile* were eligible for inclusion, although more than one diagnostic test may have been ordered for the same patient.

Demographic and clinical data collected for each specimen included age of patient (in years), gender, dates of illness onset and specimen collection, chief complaint (International Classification of Diseases, Clinical Modification [ICD-9 CM] code), and diagnostic tests that were performed and their results. De-identified demographic and clinical data and results of laboratory testing were sent to the Centers for Disease Control and Prevention (CDC), Atlanta, GA, USA for analysis. Data and specimens were anonymized and contained no identifying information that could be linked to the patient. This study was determined by CDC human subjects review to be non-research public health practice and thus did not require institutional review board review.

### Laboratory Testing

Routine diagnostics on specimens collected among KP Northwest members were performed at the KP Northwest microbiology laboratory for *Campylobacter*, *Shigella*, *Salmonella*, and *E*. *coli* O157:H7 and for the presence of Shiga toxins 1 and 2. In addition, this laboratory tested some specimens for ova and parasites and for the presence of *C*. *difficile* toxin and glutamate dehydrogenase (GDH). Tests with mixed results were reflex-tested for toxin genes using PCR. Specimens collected among KP Mid-Atlantic members were tested at the KP Mid-Atlantic microbiology laboratory for *Campylobacter*, *Shigella*, *Salmonella*, and *Yersinia enterocolitica*, as well as ova and parasites, and *C*. *difficile* toxin A and B by EIA in some specimens.

Specimens were tested for viral AGE pathogens at the Oregon State Public Health and Maryland Department of Health and Mental Hygiene laboratories. Viral nucleic acid was extracted using the KingFisher MagMax 96 viral RNA extraction kit (Life Technologies, Carlsbad, CA, USA). All specimens were tested for the presence of norovirus, sapovirus, astrovirus, and rotavirus RNA by using the AgPath-ID One-Step RT-PCR Kit (Applied Biosystems, Foster City, CA, USA) on the 7500 Realtime PCR platform (Applied Biosystems) [13–16].

### Disease Incidence Calculations

Each KP site provided the total population catchment (e.g., membership) to be used as the denominator for incidence calculations. Additionally, each KP site identified the total number of stool specimens that were submitted for diagnostic testing and the total number of patients with AGE presenting for care during the study period. AGE diagnoses for patients presenting to care were extracted and defined using a previously described set of ICD9-CM codes [17].

Pooled data on self-reported healthcare utilization practices of persons with AGE that were obtained from the Foodborne Diseases Active Surveillance Network (FoodNet) population surveys in 2000–2001, 2002–2003, and 2006–2007 [7]. These population-based telephone surveys were conducted in selected sites located throughout the United States, including Oregon, Washington, and Maryland by using a probability sample design. Data from these surveys were used to estimate healthcare-seeking rates among KP members with AGE in order to derive incidence estimates at the community level, including those that do not seek outpatient care.

**Fig 1:**
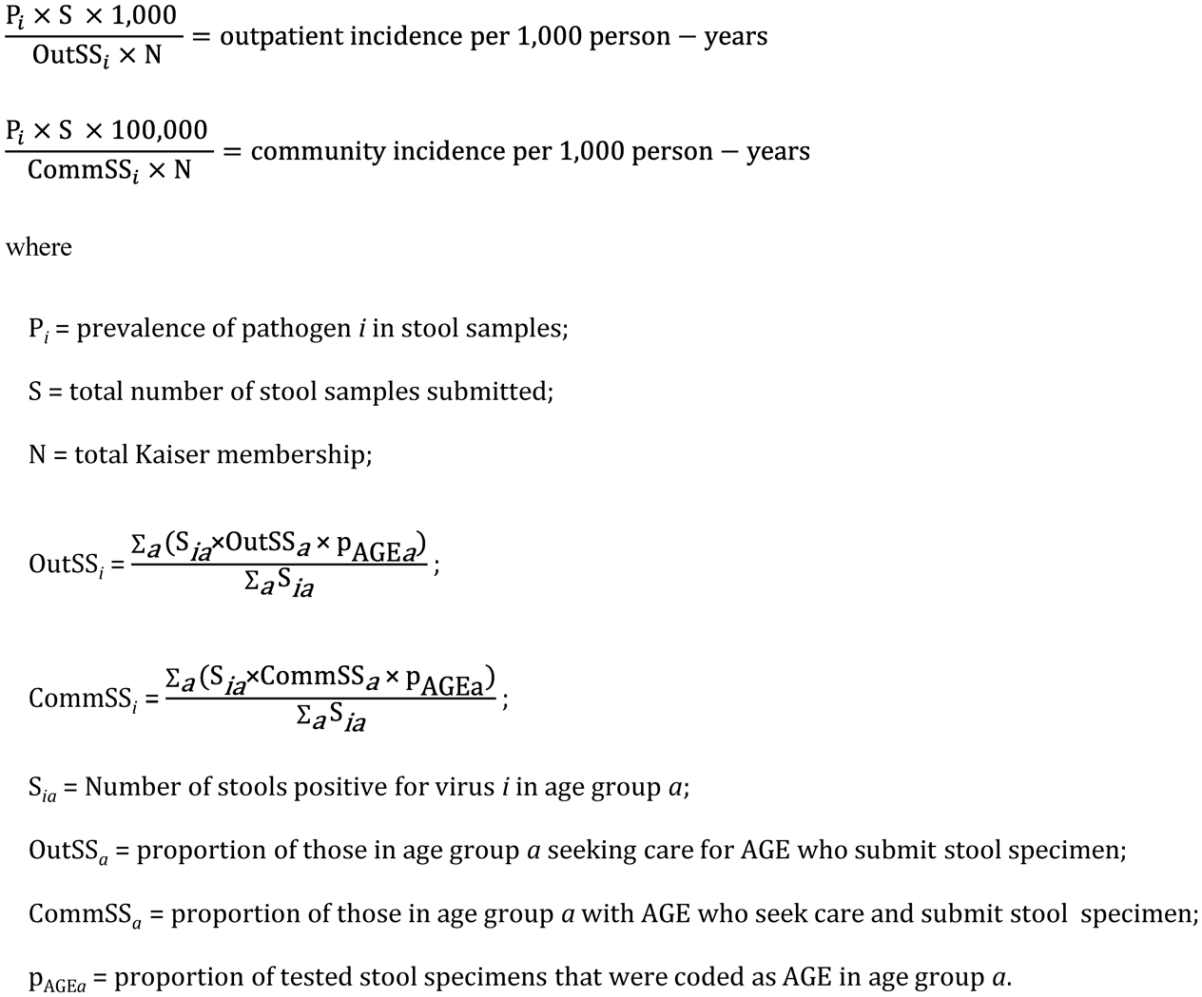
Equations used for calculation of community and outpatient incidence of viral pathogen in patients with acute gastroenteritis (AGE).

Community and outpatient incidence of each viral pathogen was calculated on the basis of the prevalence of that pathogen in sampled stool specimens (*P*_*i*_), age-weighted stool specimen submission rates among all respondents with AGE (*ComSS*_*i*_) and that among those seeking medical care (*OutSS*_*i*_), the total number of specimens submitted to the KP laboratories during the study period (*S*), and the total Kaiser membership at each site (N) (Fig 1). Thus, community incidence estimates were adjusted for both stool specimen submission and medical care-seeking rates, while outpatient incidence estimates were adjusted using only stool specimen submission rates. Age-specific estimates of norovirus incidence were calculated using the following age groups: <5, 5–15, 16–25, 26–45, 46–65, and >65 years. These age groups were broadly selected for clinical relevance and consistency in health care utilization rates. Incidence estimates were adjusted for age-specific variations in the proportion of stool specimens that were collected due to AGE being a chief complaint of the patient. A simulation approach was used to generate 95% credible intervals (CIs). For each pathogen or group of pathogens, *P*_*i*_, *ComSS*_*i*_, and *OutSS*_*i*_ were randomly drawn assuming a normal distribution for each, and the 2 incidence equations were recalculated. We report 2.5th and 97.5th centiles of 100,000 simulations. Analyses were performed by using SAS version 9.3 (SAS Institute, Cary, NC, USA).

## Results

A total of 1,099 specimens were randomly selected for inclusion in this study and tested for viral pathogens (Table 1). Fifty-five percent (n = 600) were collected among KP Northwest members, and 45% (n = 499) were collected among KP Mid-Atlantic members. Tested specimens were collected an average of 3 days after date of illness onset; 90% of specimens were collected within 7 days of illness onset. Fifty-seven percent (n = 630) of patients had an AGE chief complaint code when presenting for care. Routine bacterial culture was performed for 93% (n = 1018) of specimens; 51% (n = 558) were tested for *C*. *difficile* and 30% (n = 326) were tested for ova and parasites. Adult patients 46–65 years of age provided 33% (n = 360) of the specimens tested. One or more non-viral pathogens that cause AGE were found in 117 (11%) specimens, of which 68 (58%) were obtained from KP Northwest patients and 49 (42%) were obtained from KP Mid-Atlantic patients. Of these non-viral pathogens, patients were most commonly infected with *C*. *difficile* (n = 49, 9% of specimens tested). In addition, 32 (3%) of tested patients had a positive bacterial culture and 26 (8%) had evidence of ova or parasites.

**Table 1:** Characteristics of randomly selected stool specimens submitted by outpatients, by age group, to Kaiser Permanente Northwest and Mid-Atlantic Health Plans, 2012–2013.

Stool Specimen Characteristic	Age group, y, no. (%) positive						Total, n = 1099
	<5 years, n = 68	5–15 years, n = 72	16–25 years, n = 98	26–45 years, n = 250	46–65 years, n = 360	>65 years, n = 251	
Tested by bacterial culture	65 (96)	70 (97)	85 (87)	228 (91)	333 (93)	237 (94)	1018 (93)
Tested for C. difficile	17 (25)	13 (18)	45 (46)	111 (44)	211 (59)	155 (62)	552 (50)
Tested for ova and parasites	18 (26)	27 (38)	33 (34)	82 (33)	109 (30)	57 (23)	326 (30)
Total norovirus-positive	8 (12)	4 (6)	4 (6)	13 (5)	22 (6)	12 (5)	63 (6)
Norovirus GI	1 (13)	1 (25)	0	3 (23)	0	3 (25)	8 (13)
Norovirus GII	7 (87)	3 (75)	4 (100)	10 (77)	22 (100)	9 (75)	55 (87)
Sapovirus-positive	1 (1)	2 (3)	3 (3)	5 (2)	7 (2)	1 (0.4)	19 (2)
Astrovirus-positive	1 (1)	0	1 (1)	2 (1)	3 (1)	0	7 (1)
Rotavirus-positive	2 (3)	1 (1)	1 (1)	4 (2)	7 (2)	7 (3)	22 (2)

**Fig 2:**
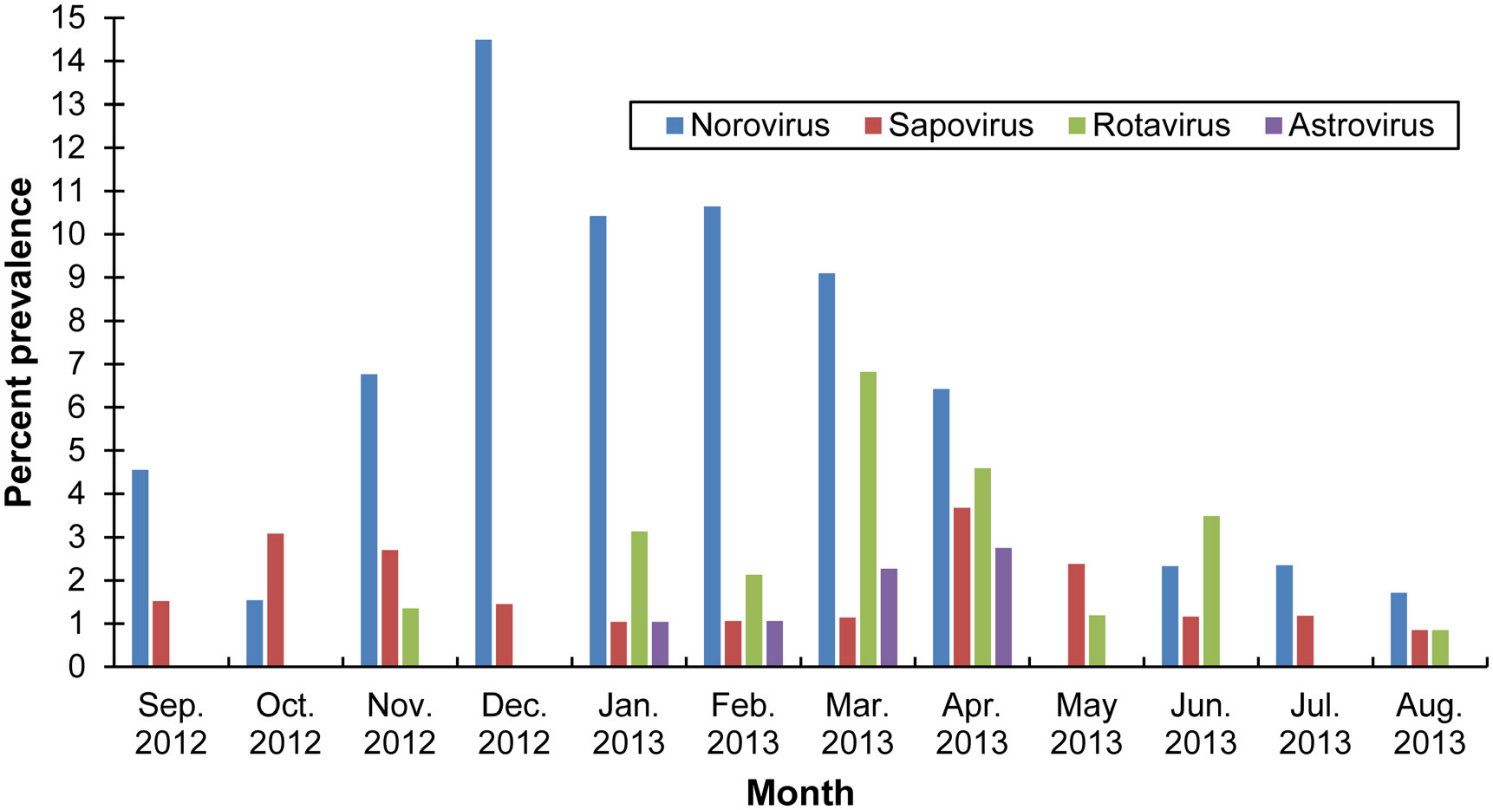
Viral pathogen prevalence in acute gastroenteritis outpatients by season, Kaiser Permanente Northwest and Mid-Atlantic Health Plans, September 2012–August 2013.

Noroviruses were the most common viral pathogen identified among patients with AGE; 63 specimens (6%) were positive for norovirus (Table 1). Additionally, 22 (2%) of specimens were positive for rotavirus; 19 (2%) were positive for sapovirus; and 7 (1%) were positive for astrovirus. Three percent of children under 5 years of age with AGE were rotavirus-positive, as were 3% of adults over 65 years of age. Patients aged 5–15 years and 16–25 years of age were most commonly infected with sapoviruses (3% among each age category). Three (43%) of the seven patients who were positive for astrovirus infection were aged 46–65 years. Eighty-six percent (n = 55) of norovirus-positive specimens were classified as genogroup (G) II. The prevalence of norovirus-positive stool specimens was highest between November and March (Fig 2). The prevalence of sapovirus-, astrovirus-, and rotavirus-positive specimens were highest during March and April.

**Fig 3:**
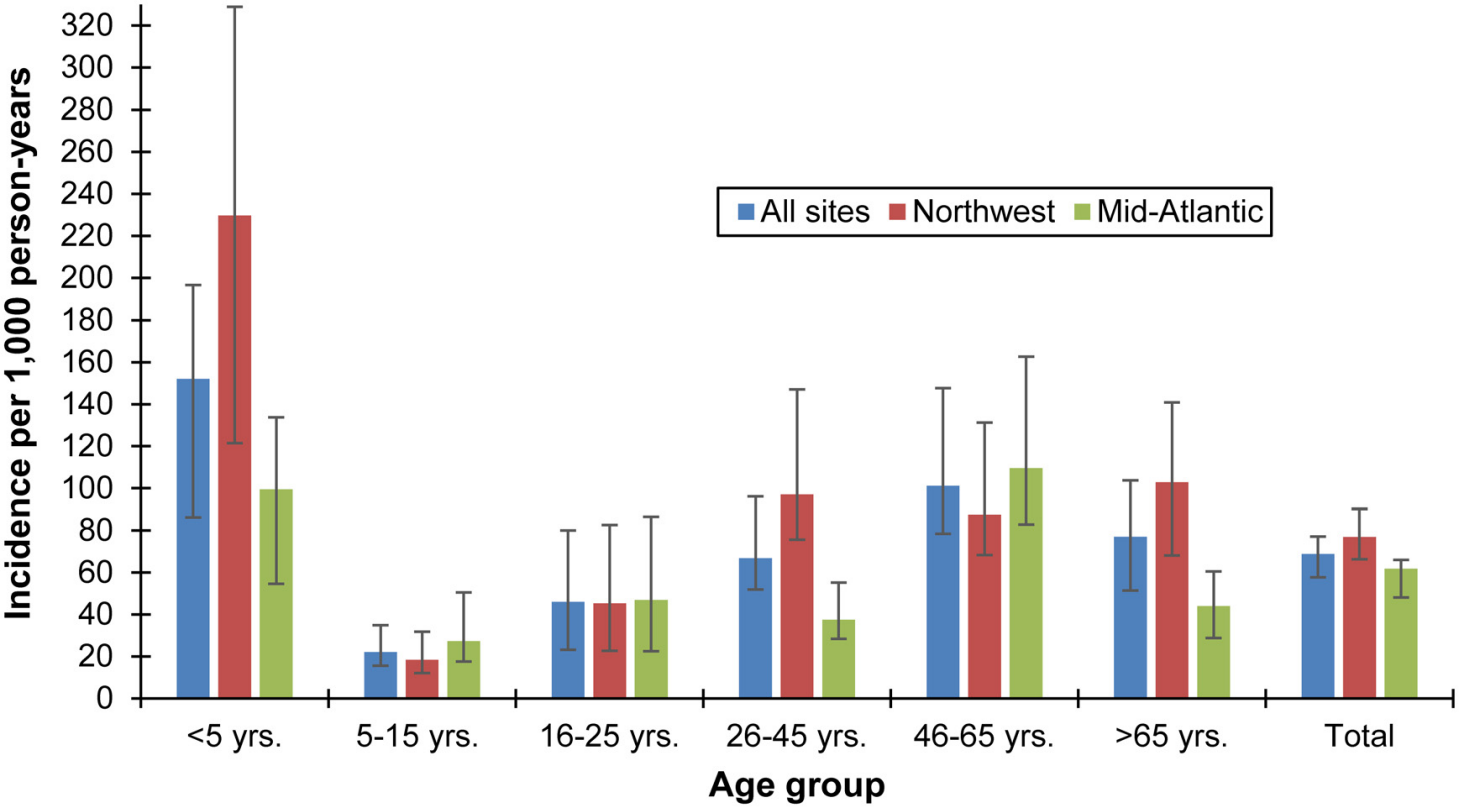
Estimated community incidence of norovirus AGE among outpatients in Kaiser Permanente Northwest and Mid-Atlantic Health Plans, by age group, 2012–2013.

**Fig 4:**
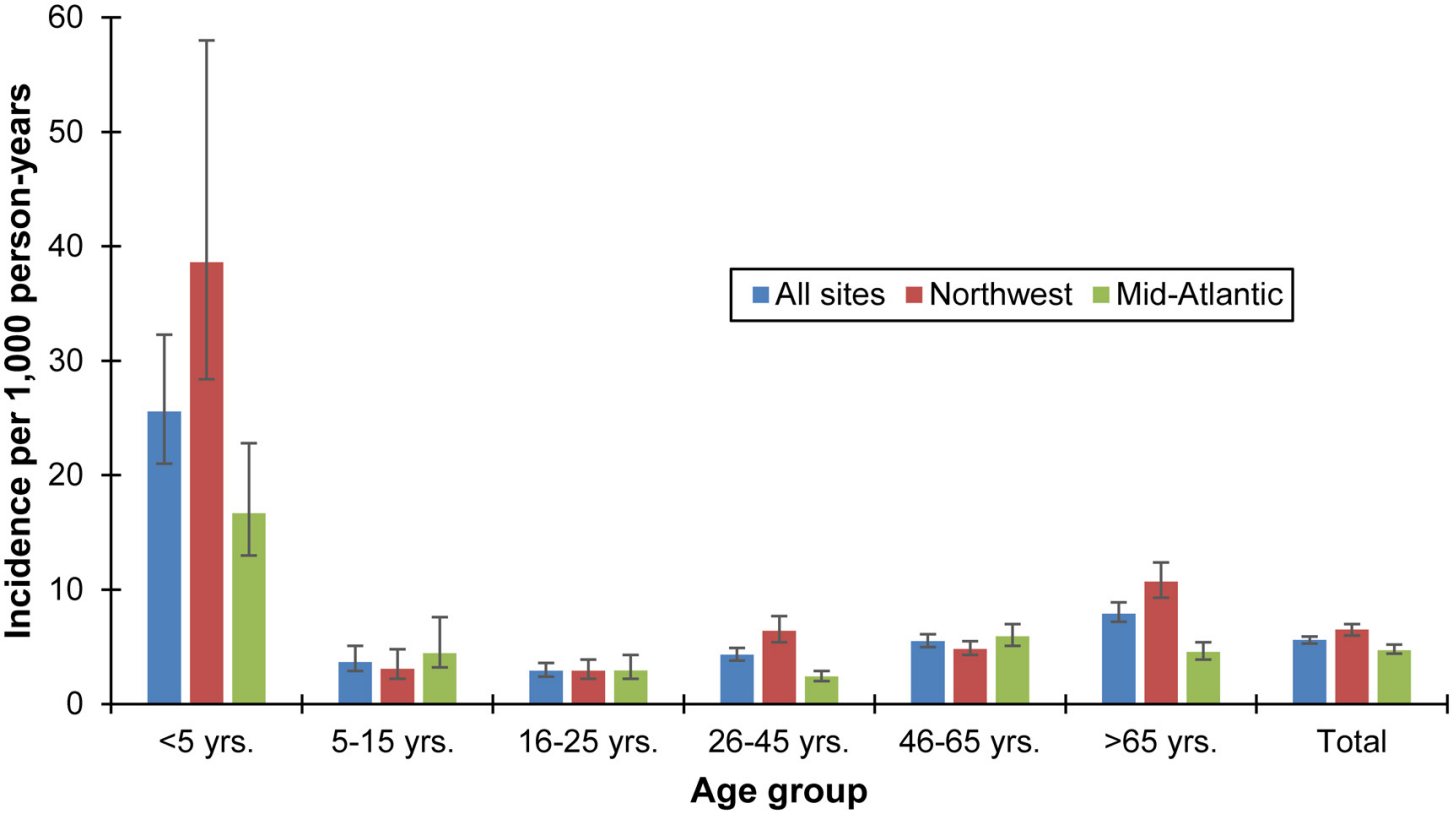
Estimated incidence of norovirus AGE among outpatients in Kaiser Permanente Northwest and Mid-Atlantic Health Plans, by age group, 2012–2013.

A total of 53,521 unique patient encounters with ICD-9CM codes consistent with AGE were identified during the study period (September 2012–September 2013 for KP Northwest, and August 2012–July 2013 for KP Mid-Atlantic), during which 6,038 stool specimens were submitted for routine clinical diagnostics (see S1 Table). Using these site-specific data along with FoodNet population survey healthcare seeking rates, the overall community incidence of norovirus-associated AGE was 68.9 per 1,000 person-years (95% credible interval: 57.7, 77.1 per 1,000) (Fig 3), while the overall incidence of norovirus-associated AGE among outpatients was 5.6 per 1,000 person-years (95% credible interval: 5.3, 5.9 per 1,000) (Fig 4). Norovirus-associated AGE incidence among KP Northwest members (community incidence: 76.9 per 1,000 person-years; outpatient incidence: 6.5 per 1,000 person-years (95% credible interval: 6.0, 7.0 per 1,000)) was similar to that among KP Mid-Atlantic members (community incidence: 61.8 per 1,000 person-years; outpatient incidence: 4.7 per 1,000 person-years). Norovirus-associated AGE incidence in the community was highest among children less than five years of age (152.1 per 1,000 person-years), followed by persons 46–65 years of age (101.2 per 1,000 person-years). Incidence of outpatient norovirus-associated AGE was also highest among children less than five years of age (25.6 per 1,000 person-years) followed by persons over 65 years of age (7.9 per 1,000 person-years).

Sapovirus-associated AGE incidence in the community was estimated at 22.7 per 1,000 person-years (95% credible interval: 17.4, 23.2 per 1,000), while incidence of sapovirus-associated outpatient visits was estimated at 1.6 per 1,000 person-years (95% credible interval: 1.6, 1.8 per 1,000) (Table 2). Sapovirus incidence was higher among KP Mid-Atlantic members when compared to KP Northwest members (community incidence: 28.6 vs. 17.8 per 1,000 person-years, respectively; outpatient incidence: 2.0 vs. 1.3 per 1,000 person-years, respectively). Rotavirus-associated AGE community incidence was estimated at 23.7 per 1,000 person-years (95% credible interval: 20.2, 26.9 per 1,000), while outpatient incidence was estimated at 1.9 per 1,000 person-years (95% credible interval: 1.9, 2.1 per 1,000). Site-specific rotavirus AGE incidence estimates were similar. Astrovirus-associated AGE community incidence was estimated at 8.5 per 1,000 person-years (95% credible interval: 6.4, 8.6 per 1,000), while outpatient incidence was estimated at 0.6 per 1,000 person-years (95% credible interval: 0.6, 0.7 per 1,000). Astrovirus incidence was roughly three times higher among KP Mid-Atlantic members (Table 2). Age-specific incidence estimates for sapovirus, rotavirus, and astrovirus AGE were not calculated due to the relatively low prevalence of each virus among submitted specimens in some age strata.

**Table 2:** Outpatient and community incidence of viral pathogens that cause AGE, by site.

Virus	Outpatient incidence per 1,000 person-years (95% credible interval)			Community incidence per 1,000 person-years (95% credible interval)		
	Northwest	Mid-Atlantic	Both Sites	Northwest	Mid-Atlantic	Both Sites
Norovirus	6.5 (6.0, 7.0)	4.7 (4.4, 5.2)	5.6 (5.3, 5.9)	76.9 (66.3, 90.1)	61.8 (48.1, 65.8)	68.9 (57.7, 77.1)
Sapovirus	1.3 (1.2, 1.4)	2.0 (1.9, 2.3)	1.6 (1.6, 1.8)	17.8 (12.9, 17.5)	28.6 (21.4, 29.2)	22.7 (17.4, 23.2)
Astrovirus	0.4 (0.3, 0.4)	0.8 (0.8, 1.0)	0.6 (0.6, 0.7)	3.8 (3.7, 5.0)	12.8 (8.9, 12.2)	8.5 (6.4, 8.6)
Rotavirus	2.1 (1.8, 2.1)	1.9 (1.8, 2.1)	1.9 (1.9, 2.1)	21.1 (20.3, 27.5)	26.5 (19.6, 26.8)	23.7 (20.2, 26.9)

## Discussion

This study represents an important advancement in norovirus epidemiology by providing the first U.S. laboratory-based age-specific norovirus AGE incidence rates in both community and outpatient settings. This study is also one of few to report the incidence of AGE associated with rotavirus, astrovirus, and sapovirus across the full age spectrum at the community and outpatient levels. Approximately 6% of specimens submitted for routine bacterial culture at the two KP study sites were positive for norovirus, and the highest norovirus prevalence was identified among specimens collected from children less than 5 years of age, consistent with previous findings from a similar study [7]. Furthermore, the prevalence of viral pathogens that cause AGE were highest in the winter and early spring months, consistent with previous descriptions of norovirus and rotavirus seasonality in temperate climates [18, 19]. A very high proportion of norovirus-positive specimens were genogroup GII, as expected from earlier reports [10, 20, 21].

Our all-ages norovirus incidence estimates for outpatient (5.6 per 1,000 person-years) and community (68.9 per 1,000 person-years) settings are consistent with earlier studies in the U.S. and other developed countries. Our results are very similar to the outpatient and community norovirus incidence of 6.4 cases and 65 cases per 1,000 person-years, respectively as reported by Hall et al for a KP population in Georgia [7]. Gastanaduy et al. [22] reported a similar U.S. outpatient incidence rate of norovirus AGE at 5.7 per 1,000 and Scallan et al. [23] reported a similar U.S. community norovirus incidence of 70 per 1,000. Our estimated outpatient incidence estimates are similar to those reported in Germany (6.3 per 1,000 person-years) [24] and England (5.4 per 1,000 person-years) [11]. In contrast, our outpatient incidence estimate was much lower than one estimate reported from The Netherlands (9.2 per 1,000) [25], but also much higher than a similar study conducted in the United Kingdom (2.1 per 1,000) [26]. Our community incidence estimate is somewhat similar to estimates reported by two studies in the UK (45–47 per 1,000) [11, 26], while community incidence reported by a Dutch study was lower (38 per 1,000) [25] and a Canadian community incidence estimate was much higher (104 per 1,000) [27]. Differences in healthcare systems and healthcare utilization practices likely account for at least some of the observed differences in norovirus incidence among these countries.

Some of our age-specific norovirus incidence estimates are consistent with age-specific estimates reported from one study in the United Kingdom [11]. Our age-specific outpatient incidence estimates among children under 5 years (25.6 per 1,000) and children aged 5–15 years (3.7 per 1,000) are similar to the United Kingdom incidence estimates for children under 5 years of age (32 per 1,000) and 5–14 years of age (4.4 per 1,000). Conversely, our outpatient incidence rates for older adults (aged 46–65 years: 5.5 per 1,000; over age 65 years: 7.9 per 1,000) were at least twice the outpatient incidence rates reported among these similar age groups (45–64 years: 2.6 per 1,000; 65 years of age and older: 3.7 per 1,000). Differences in age categories used between these studies limit our ability to compare community incidence rates across the two studies; however, our age-specific incidence community incidence rates are markedly lower among children (less than 5 years of age: 152.1 per 1,000; aged 5–15 years: 22.2 per 1,000) than among children participating in the UK study (less than 5 years of age: 214 per 1,000; 5–14 years of age: 65 per 1,000).

As expected, the prevalence and estimated incidence of norovirus were highest among children less than 5 years of age in our study. Our estimated outpatient incidence of norovirus among children less than 5 years of age is similar to that developed through indirect regression of all-cause AGE from a U.S. insurance claim database [22] as well as that from direct active surveillance in pediatric clinics [9]. Interestingly, the outpatient and community incidence of norovirus among Kaiser Permanente Northwest members less than 5 years of age participating in were approximately twice as high as those among Kaiser Permanente Mid-Atlantic members in this age group. This is despite the fact that the proportion of persons less than 5 years of age who submitted a stool specimen of those with AGE who sought medical care was higher among Kaiser Permanente Mid-Atlantic members (5.3%) than among KP Northwest members of the same age group (3.8%).

Our estimates of sapovirus incidence (community: 22.7 per 1,000 person-years; outpatient: 1.6 per 1,000 person-years) are somewhat higher than those reported in the previous Georgia Kaiser Permanente study (community: 9.0 per 1,000 person-years; outpatient: 1.1 per 1,000 person-years) [7], though the credible intervals of the respective estimates were still overlapping. In contrast, our estimated astrovirus incidence rates (community: 8.5 per 1,000 person-years; outpatient: 0.6 per 1,000 person-years) are substantially lower than those from the Georgia study (community: 18 per 1,000 person-years; outpatient: 2.7 per 1,000 person-years) [7]. Our estimated community rotavirus incidence rate (23.7 per 1,000 person-years) is also higher than that reported among specimens taken from Georgia KP members in 2004–2005 (8.8 per 1,000 person-years), although our estimated outpatient rotavirus incidence rate (1.9 per 1,000 person-years) is similar to the earlier report (1.5 per 1,000 person-years). These observed differences in incidence between the two studies may be due in part to regional or year-to-year variations.

The principal limitation of this study results from using routinely collected fecal specimens for bacterial diagnostics to assess the incidence of viral pathogens that cause AGE. Active systematic collection of specimens from all patients presenting with AGE would likely provide more accurate incidence estimates. In addition to potentially biasing towards bacterial etiologies (and thus away from viruses), this sampling approach may have also included some patients with noninfectious or chronic causes of AGE; this has been attributed as a possible reason for the low detection of norovirus in specimens from similar studies [7, 10]. Indeed, many specimens were included in our study from patients without an AGE-specific ICD-9CM code, although ICD coding accuracy has been shown to be suspect [28]. Additionally, given the potential for viral pathogens to cause asymptomatic infections among healthy individuals, detection of these viruses among patients with AGE may not always indicate etiology; however a multi-center case control study of pediatric AGE in the U.S. found these viruses in <5% of healthy controls [6, 9]. A limitation of our community incidence estimates is the use of somewhat dated national healthcare seeking rates that are based on diarrheal disease. The healthcare seeking rates from the FoodNet population surveys did include respondents from geographic areas in our study, but no site-specific healthcare seeking rates were available. In addition, the use of healthcare utilization information based on diarrheal disease may lead to an underestimate of viral pathogens such as norovirus that can cause other signs of AGE (e.g., vomiting) in the absence of diarrhea. Furthermore, more severe cases of AGE are presumably more likely to seek care and could bias our results toward increased prevalence of pathogens that cause more severe symptoms. Finally, our incidence estimates are limited to two metropolitan populations. While these two populations are on opposite sides of the United States, the incidence estimates that we have generated cannot be generalized to the United States population.

Population-based surveillance for laboratory-confirmed AGE due to viral pathogens provides the most direct assessment of disease incidence, despite limitations in generalizability. The age-specific incidence rates that we have reported serves to identify groups most often infected and assists in focusing future prevention efforts such as administration of possible future vaccines. Surveillance platforms that encompass larger populations over longer periods of time are needed to provide trends in the incidence of AGE due to viral pathogens that are generalizable to the entire population. Our demonstrated burden of norovirus AGE in the community and in outpatient settings justifies the effort to develop possible norovirus vaccines.

## Supporting Information

S1 Table Healthcare utilization information among persons with acute gastroenteritis (AGE) used for incidence calculations, by age group. (DOCX)
