# Supplementary material for: Incidence of Norovirus and Other Viral Pathogens That Cause Acute Gastroenteritis (AGE) among Kaiser Permanente Member Populations in the United States, 2012–2013
Source: PLoS One. 2016 Apr 26;11(4):e0148395. doi: 10.1371/journal.pone.0148395 (PMC4846013; doi:10.1371/journal.pone.0148395)
Supplement: S1 Table — (DOCX) [file pone.0148395.s001.docx]

**S1 Table. Healthcare utilization information among persons with acute gastroenteritis (AGE) used for incidence calculations, by age group.**

| **Characteristic** | **Age group** | | | | | | **Total** |
| --- | --- | --- | --- | --- | --- | --- | --- |
|  | **<5 years** | **5-15 years** | **16-25 years** | **26-45 years** | **46-65 years** | **>65 years** |  |
| % Persons with AGE who sought medical care (Source: FoodNet Population Survey) | 20.7 | 16.8 | 7.7 | 6.3 | 5.3 | 11.5 | 8.5 |
| Estimated population catchment: |  |  |  |  |  |  |  |
| Northwest | 22,097 | 61,508 | 62,624 | 120,263 | 139,288 | 69,815 | 475,595 |
| MId-Atlantic | 19,983 | 58,654 | 72,886 | 121,219 | 149,080 | 60,418 | 482,240 |
| Total | 42,080 | 120,162 | 135,510 | 241,482 | 288,368 | 130,233 | 957,835 |
| AGE-coded patients presenting for care (%) |  |  |  |  |  |  |  |
| Northwest | 2729 (12) | 1803 (3) | 2602 (4) | 6152 (5) | 8724 (6) | 7944 (11) | 29,954 (6) |
| MId-Atlantic | 2782 (14) | 1886 (3) | 2231 (3) | 4800 (4) | 6108 (4) | 5760 (10) | 23,567 (5) |
| Total | 5511 (13) | 3689 (3) | 4833 (4) | 10,952 (5) | 14,832 (5) | 13,704 (11) | 53,521 (6) |
| Stool specimens submitted for routine diagnostics (%) |  |  |  |  |  |  |  |
| Northwest | 176 (7) | 147 (8) | 222 (9) | 575 (9) | 872 (10) | 638 (8) | 2630 (9) |
| MId-Atlantic | 242 (8) | 193 (10) | 348 (16) | 870 (18) | 1075 (18) | 680 (12) | 3408 (15) |
| Total | 418 (8) | 340 (9) | 570 (12) | 1445 (13) | 1947 (13) | 1318 (10) | 6038 (11) |
| Stool specimens selected and tested for viral pathogens |  |  |  |  |  |  |  |
| Northwest | 27 (15) | 41 (28) | 52 (23) | 133 (23) | 190 (22) | 157 (25) | 600 (23) |
| MId-Atlantic | 41 (17) | 31 (16) | 46 (13) | 117 (13) | 170 (16) | 94 (14) | 499 (15) |
| Total | 68 (16) | 72 (21) | 98 (17) | 250 (17) | 360 (19) | 251 (19) | 1099 (18) |
| % of tested specimens contain AGE as chief complaint |  |  |  |  |  |  |  |
| Northwest | 16 (59) | 19 (46) | 29 (56) | 72 (54) | 117 (62) | 96 (61) | 349 (58) |
| MId-Atlantic | 25 (61) | 14 (45) | 21 (46) | 66 (56) | 90 (53) | 63 (67) | 279 (56) |
| Total | 41 (60) | 33 (46) | 50 (51) | 138 (55) | 207 (58) | 159 (63) | 628 (57) |
